# Nurse Adaptability and Post-traumatic Stress Disorder Symptoms During the COVID-19 Pandemic: The Effects of Family and Perceived Organizational Support

**DOI:** 10.3389/fpsyg.2021.749763

**Published:** 2022-03-04

**Authors:** Mona Cockerham, Margaret E. Beier, Sandy Branson, Lisa Boss

**Affiliations:** ^1^School of Nursing, Sam Houston State University, The Woodlands, TX, United States; ^2^Department of Psychological Sciences, Rice University, Houston, TX, United States; ^3^Cizik School of Nursing, The University of Texas Health Science Center, UT-Health, Houston, TX, United States

**Keywords:** Coronavirus, nurses, PTSD, family support, perceived organizational support, adaptability

## Abstract

**Objective:**

To examine the effect of family and perceived organizational support on the relationship between nurse adaptability and their experience with COVID-related PTSD (post-traumatic stress disorder) symptoms in frontline nurses working on COVID-19 units.

**Background:**

Proximity to and survival of life-threatening events contribute to a diagnosis of PTSD, which is characterized by avoidance of reminders of trauma, intrusive thoughts, flashbacks of events, sleep disturbances, and hypervigilance. Using the job-demands and resource model, we examined the effect of adaptability, family support, and perceived organizational support on PTSD symptoms for nurses during the COVID-19 pandemic. Specifically, we tested whether perceptions of environmental supports—i.e., family and organizational support—moderated the relationship between nurse adaptability and COVID-related PTSD symptoms.

**Methods:**

A sample of frontline nurses working on COVID-19 units during the COVID-19 pandemic in Texas (*N* = 277) participated in this cross-sectional, observational study. Frontline Nurses reported demographic information and completed surveys designed to measure adaptability, perceived organizational support, family support, and COVID-related PTSD symptoms.

**Results:**

Adaptability was significantly positively correlated (medium effects) to perceived organizational and family support (*r* = 0.51 and 0.56, respectively, *p* < 0.01). Adaptability and perceived organizational support were also negatively correlated with COVID-related PTSD symptoms (medium effects). Adaptability was negatively correlated with COVID-related PTSD symptoms, supporting Hypothesis 1 (*r* = −0.43, *p* < 0.01). Perceived organizational support was also significantly negatively correlated with COVID-19-related PTSD symptoms (*r* = −0.30, *p* < 0.01). Family support was not significantly correlated with COVID-related PTSD but was positively related to experiencing COVID-related PTSD after other variables were accounted for.

**Conclusion:**

Findings suggest that individual adaptability and organizational support may reduce PTSD severity in frontline nurses working during a crisis; however, family support may increase PTSD symptoms. We provide suggestions for strengthening individual adaptability and healthcare leadership including remaining highly engaged to show support by providing rapid communication, remaining calm during difficult circumstances, and maintaining a consistent, physical presence during difficult times. Moreover, our results suggest additional support for nurses with families to adapt to crisis.

## Introduction

Infecting over 33 million people in the United States the mental health impact of the COVID-19 pandemic on nurses is a public health emergency (Centers for Disease Control [CDC], 2021; Texas Department of Health and Human Services [THHS], 2021). Much attention is focused on poor health outcomes and associated sequalae from a COVID-19 infection, but investigation of mental health outcomes is critically important as it relates to COVID-19 and subsequent outbreaks. However, scant evidence related to mental health outcomes and COVID-19 shows that healthcare workers and nurses most commonly reported depression, insomnia, severe anxiety, and post-traumatic stress disorder (PTSD) resulting from their experiences working on the front lines (Carmassi et al., 2020; Giorgi et al., 2020; Preti et al., 2020). For nurses specifically, perhaps the most worrisome mental health outcome related to COVID-19 is PTSD and associated symptoms (Schuster and Dwyer, 2020). During public health crises, like a pandemic, frontline health workers are among the most vulnerable group to develop mental health issues and PTSD, including nurses (Kisely et al., 2020; Sagherian et al., 2020). Traumatic events for nurses during the COVID-19 pandemic include repeated exposure to an unprecedented number of critically ill patients, high mortality rates, unpredictable course of the virus, and lack of effective treatment guidelines (Peerim et al., 2020; Wang, 2020). Additional factors directly related to the development of PTSD during a pandemic are the worry about personal health and health of loved ones, concerns about spreading disease, fear of social contact, and uncertainty related to the future course of the outbreak (Kisely et al., 2020; Busch et al., 2021). Clearly, their position as key frontline health workers puts nurses at risk for poor mental health outcomes, such as PTSD.

The purpose of this cross-sectional study was to examine the person and environment factors that affect PTSD symptoms in the context of highly stressful health crises for nurses. To understand the impact of COVID-19 on the nurses’ experience in a relatively constrained environment, we focused on one state, Texas in the United States. Limiting the research site to one state permits us to more accurately describe how the pandemic was affecting nurses during the time of our study.

**FIGURE 1:**
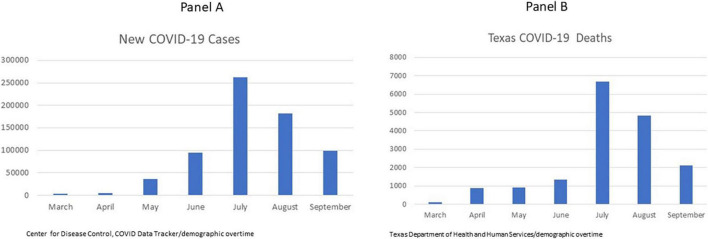
COVID-19 new cases (Panel **A**) and deaths (Panel **B**) in Texas between March and September 2020 (Wave 1). Data for this study were collected from March through July, August, and September 2020 are shown for comparison.

Figure 1 shows the situation in Texas between March and July, 2020, when these data were collected (August and September, 2020, are included for comparison purposes). During these months, frontline healthcare workers faced changes in their workflow and patient care protocols along with social media stresses on numbers of nurses dying leading to overwhelming feelings of fear, depression and stress. According to Levin (2019) in early phases of pandemics there tends not to be given to the mental consequences of working with these mental stressors.

**FIGURE 2:**
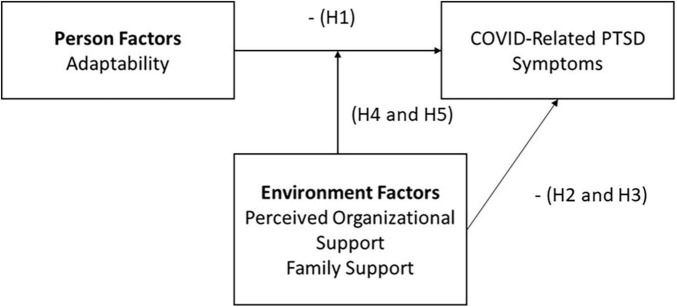
Graphical depiction of study hypotheses and variables. PTSD, post-traumatic stress disorder.

The current study integrates research and theory on adaptability (Ployhart and Bliese, 2006), job demands and resources (Demerouti et al., 2001), and family support (King et al., 1995) to examine the person and environmental factors that affect nurses’ PTSD symptoms during the COVID-19 Pandemic. We anticipated that nurses working in COVID-19 units who are higher in adaptability (a dispositional trait related to one’s ability to adjust to changing circumstances) would be less likely to report PTSD symptoms. Furthermore, the job-demands and resource theory (JDR) posits that job characteristics that negatively affect employee wellbeing (e.g., job demands that cause strain like work overload, emotional demands, physical job demands, and work-home conflict), can be offset by resources such as organizational and family support (Bakker and Demerouti, 2017). Our theoretical model is shown in Figure 2.

### Adaptability

Individual adaptability is defined as a person’s dispositional tendency to adjust him or herself to fit new tasks or environments (Ployhart and Bliese, 2006). As such, individual adaptability represents relatively stable cognitive (e.g., emotion regulation) and behavioral (e.g., active engagement and asking for help) tendencies employed by people while they face new and dynamic situations. Importantly, in this definition, adaptability is a function of the person, not the situation, although success in some situations requires more adaptability than others (Pulakos et al., 2000; Ployhart and Bliese, 2006).

Research suggests that individual adaptability is positively related to job satisfaction and job performance, and negatively related to turnover intentions for new employees (Wang et al., 2011; Cullen et al., 2014). Individual adaptability is also shown to be negatively related to general depressive symptoms (e.g., negative thoughts, distress, fear of not mattering) for students surveyed during the start of the COVID-19 pandemic (Besser et al., 2020). Although the concept of adaptability was popular in previous nursing research (Jones, 1991), current conceptualizations of trait individual adaptability have not been extensively studied with nurses. Authors of a cross-sectional study of nurses found that cognitive adaptability was negatively related to emotional exhaustion and depersonalization, and positively related to perceptions of personal accomplishment (Browning et al., 2006). Authors of studies examining dispositional tendencies related to adaptability, namely, perceiving challenge as part of everyday life and perceived control over one’s environment (Ployhart and Bliese, 2006), have also found these tendencies to be negatively related to burnout in nurses (Garrosa et al., 2010, 2011). As such, we expected that individual adaptability would be negatively related to experiencing PTSD symptoms (Hypothesis 1).

In the current study, we measured individual adaptability as a composite of four facets of dispositional adaptability: work-stress, uncertainty, crisis, and interpersonal-oriented adaptability (Pulakos et al., 2002; Ployhart and Bliese, 2006). Work-stress adaptability refers to a person’s ability to learn new ways to perform a job. Examples include remaining calm under pressure, not overreacting to unexpected situations, and demonstrating the highest level of professionalism during stressful circumstances (Pulakos et al., 2002). Uncertainty-oriented adaptability refers to the extent to which a person can effectively deal with the unpredictable nature of certain situations. Examples include effectively adjusting goals, actions, and priorities in response to changing situations, imposing structure for the team during dynamic situations, and not being paralyzed by uncertainty (Pulakos et al., 2002). Interpersonal-oriented adaptability refers to a person’s ability to effectively interact with others. Examples include being flexible and open-minded while working with a team, developing an effective relationship with diverse and differing personalities, and accepting development feedback related to work performance (Pulakos et al., 2002). Finally, crisis-oriented adaptability refers to a person’s ability to react appropriately and decisively to life-threatening or dangerous situations. Examples of adaptable behaviors in this dimension include maintaining emotional control during dangerous situations, stepping up to act in an emergency, and making split-second decisions based on clear thinking (Pulakos et al., 2002). Facets of adaptability deemed less-relevant to nursing during the COVID-19 pandemic were omitted: i.e., creativity (solving ill-defined problems creatively), learning (anticipates and learns new skills for future job requirements), cultural (performs effectively in different cultures), and physical (adjusts to difficult physical environments; Pulakos et al., 2002; Ployhart and Bliese, 2006).

### Perceived Organizational and Family Support

Organizational support theory posits that employees form a general perception about the extent to which the organization values their contributions and cares about their wellbeing, termed perceived organizational support (Eisenberger et al., 2020). These perceptions are a function of employee judgments about fairness, human resource practices, work conditions, and leadership. Employees who experience stressors affecting their ability to do their work (e.g., overload or ambiguity) are less likely to perceive that the organization supports them (Kurtessis et al., 2017). But perceptions of organizational support will be less affected when employees perceive that adverse conditions are outside of the organization’s control (e.g., pandemics, government regulations; Eisenberger et al., 2020). Perceived organizational support is linked to an array of positive outcomes such as job performance, organizational commitment, engagement, trust, and most relevant to the current discussion, employee wellbeing (Kurtessis et al., 2017; Eisenberger et al., 2020). Specifically, a meta-analysis on the relationship between perceived organizational support and different facets of wellbeing found that employees who felt supported by the organization were significantly less likely to experience stress, burnout, and emotional exhaustion (rho’s −0.43, −0.46, and −0.47, respectively).

Perceived organizational support has also been examined in the context of nursing. Researchers found that nurses who perceived organizational support experienced higher career and job satisfaction (Polat and Terzi, 2020; Türe and Akkoç, 2020), were less likely to report chronic fatigue syndrome (Li M. et al., 2020), and were less likely to intend to leave nursing (Li X. et al., 2020). Further, a study of nurses during the SARS pandemic found that perceptions of organizational support predicted lower levels of avoidance behaviors, emotional exhaustion, and nurse anger (Marjanovic et al., 2007). George et al. (1993) also found that perceived organizational support moderated the relationship between exposure to AIDS patients and nurses’ negative mood such that perceived organizational support buffers the effect of exposure to AIDS patients. Similarly, perceived support was particularly important in conditions of high stress/threat in a sample of ICU nurses (Verhaeghe et al., 2008). In the context of JDR theory (Demerouti et al., 2001), we posit that perceived organizational support acts as a job resource that reduces symptoms of PTSD for nurses. As such, we expected a negative overall relationship between PTSD and perceived organizational support (Hypothesis 2).

In addition to perceived organizational support (Eisenberger et al., 2020), family support is related to employee wellbeing. Family support includes emotional (e.g., talking to a spouse about a difficult day) and instrumental assistance (e.g., helping with day-to-day task related support, which influences the family or home operations; King et al., 1995; French et al., 2018). Family support is linked to positive outcomes such as health (Uchino et al., 1996), reductions in work-family conflict (French et al., 2018), and increased job satisfaction (Ford et al., 2007). Findings from a study of frontline hospitality workers during COVID-19 also showed that perceived family support mitigated the effect of COVID-related layoffs on perceived stress (Tu et al., 2021). Additionally, authors of two separate research studies during and/or after the SARS outbreak in 2003 reported that greater family support positively influenced the health and wellbeing of frontline health workers (Sin and Huak, 2004) and was also significantly associated with decreased levels of anxiety, depression, and sleep quality of nurses (Chen et al., 2006). Thus, aligned with the JDR theory (Demerouti et al., 2001), we expected that nurses who report more family support will report fewer PTSD symptoms (Hypothesis 3).

In addition to the main effects of perceived organizational support and family support on COVID-related PTSD symptoms, we anticipated that these factors would moderate the relationship between adaptability and COVID-related PTSD symptoms. Similar to past research findings that perceptions of support were particularly important in conditions of high threat and strain (George et al., 1993; Verhaeghe et al., 2008), we anticipated that nurses lower in adaptability, and thus more likely to experience strain due to the pandemic, would benefit more from perceived organizational support compared with nurses who are already relatively high in adaptability (Hypothesis 4). Similarly, we expected that nurses lower in adaptability would benefit most from family support compared with nurses who are relatively higher in adaptability (Hypothesis 5).

Findings from the current study contribute to the literature in four ways. First, although researchers have recorded the distress experienced by nurses during this and other pandemics (Busch et al., 2021) and determinants of PTSD symptoms such as family status, availability of personal protective equipment (PPE), and years of experience (Kisely et al., 2020), limited research has examined dispositional traits, such as adaptability, on nurse stress and burnout. Second, we integrate the job demands and resources model (Demerouti et al., 2001) and adaptability theory to explain how resources, like perceived organizational and family support, moderate the relationship between nurse adaptability and a health-related outcome (PTSD) for nurses. Third, we examine the influence of both family and perceived organizational support to understand how resources from different life domains might mitigate the effects of the pandemic on nurses’ PTSD symptoms. Because the pandemic clearly affected nurses in both work and family domains (e.g., worry about infecting family members; Kisely et al., 2020), we thought it particularly important to examine the effect of both on PTSD symptoms. Fourth our study points to levers that hospitals can use to improve nurses’ mental health during pandemics.

## Materials and Methods

### Participants and Procedure

Nurses were recruited through the Texas Nurses Association’s membership and snowball recruitment email method by nurses forwarding the recruitment email to other nurses and through social media. Limiting the research site to one state permits us to more accurately describe how the pandemic was affecting nurses during the time of our study. The recruitment email provided a link for nurses to complete an electronic survey (Qualtrics) and participants were compensated with a $5 gift card for completing all study questions.

Data were collected in May through July, 2020. After providing consent, nurses completed a 165-item survey (additional measures as part of a larger study are not reported here). Demographic data (age, gender, job title, hospital city/state location, years of experience in current hospital, and education) was collected and nurses were asked if they worked in a COVID-19 specialty unit or designated unit. A total of 945 nurses completed at least part of the survey, of those we eliminated from further consideration nurses who did not complete at least 90% of the survey and nurses who spent less than 600 s (10 min) or more than 10,000 s (166 min) on the survey, those who did not work in Texas (26 nurses), and those who did not work on a COVID-19 unit. The time constraints were a conservative control for careless responding given that we estimated a generous 30 min for taking the survey in pilot testing. After removing participants based on the time constraints, the average time for survey completion was 24 min. The final sample included *N* = 277 nurses (*N* = 229 women), with 29% of those who started the survey being included in our sample. Mean years of experience at the current hospital was 5.54 years (SD, 3.12), mean age was 31.98 years (SD 5.89).

### Measures

#### Adaptability

The I-ADAPT-M (Ployhart and Bliese, 2006) was used to measure (1) crisis-oriented adaptability (sample item is “I am able to maintain focus during emergencies,” 6 items, α = 0.85), work stress-oriented adaptability (sample item is “I am usually stressed when I have a large workload,” 5 items, α = 0.85), interpersonal-oriented adaptability (sample item is “I am an open-minded person in dealing with others,” 5 items, α = 0.80), and uncertainty-oriented adaptability (sample item is “When something unexpected happens I readily change gears in response,” 6 items, α = 0.70). Participants responded on a 7-point Likert scale where *1* = *strongly disagree* and *7* = *strongly agree*. We conducted a confirmatory factor analysis to support the creation of a composite measure of adaptability. We modeled a single latent factor from the four adaptability variables as described above using the lavaan package in R (Rosseel, 2012). Fit of this model was good, χ^2^ (2, 277) = 2.583, *p* > 0.01, comparative fit index (CFI) = 0.99, root mean square error of approximation (RMSEA) = 0.028, where models with CFI greater than 0.95 and RMSEA less than 0.05 are considered good fit (Bollen, 1989). Thus, we created a composite of the adaptability measure for further analysis by summing and averaging scores from each participant.

#### Perceived Organizational Support

We used an eight-item measure of perceived organizational support (Eisenberger et al., 1986). Sample items are, “The organization really cares about my wellbeing,” and “The organization fails to appreciate any extra effort from me” (reverse coded). Items were rated on a seven-point Likert scale where *1* = *strongly disagree* and *7* = *strongly agree* (α = 0.84).

#### Family Support

Six items from the King et al. (1995) family support inventory for workers were used to assess family support. Three items focused on instrumental assistance (e.g., “My family members do their fair share of household chores”) and three on emotional support (e.g., “When I succeed at my work, members of my family show that they are proud of me”). Items were rated on a five-point Likert scale where *1* = *strongly disagree* and *5* = *strongly agree*. Item analysis revealed that one of the instrumental assistance items was not correlated with the others (i.e., “My family leaves too much of the daily details of running the house to me”) even after reverse coding. We thus formed composites with two items for instrumental assistance and three items for emotional support subscales. Because these two subscales were correlated (*r* = 0.50, *p* < 0.01) and because we had no theoretical reason to separate them in the current study, we created a composite with all five family support items. Notably, other research has also found that emotional and instrumental family support overlap considerably (French et al., 2018). The internal consistency reliability estimates for the family support composite, which is used in all subsequent analyses, was α = 0.73.

#### COVID-Related Post-traumatic Stress Disorder

The 17-item civilian version of the PTSD Checklist (PCL-C; Weathers et al., 2013) was used to assess COVID-related PTSD severity (McDonald and Calhoun, 2010). According to the American Psychiatric Association [APA] (2013), DSM spells this out: “PTSD is psychiatric disorder that may occur in people who have experienced or witnessed a traumatic event such as a natural disaster, a serious accident, a terrorist act, war/combat, or rape or who have been threatened with death, sexual violence or serious injury.” Respondents were instructed to indicate the extent to which they have been bothered by a symptom in the last month since the outbreak of COVID-19 using a 5-point Likert-type scale where *1* = *not at all to 5* = *extremely*. A sample item is “Repeated, disturbing memories, thoughts, or images of a stressful experience from the past” (α = 0.94).

#### Analytical Strategy

Composites for multi-item constructs were created by averaging scores across individual items within a scale. A correlational and regression approach was used to test the hypotheses in R (R Core Team, 2021). Specifically, hierarchical moderated regression was conducted to examine the independent variance accounted for in PTSD symptoms by each of the predictors and the interactions.

## Results

Descriptive statistics and correlations for all variables are shown in Table 1. Adaptability was significantly positively related to perceived organizational and family support (*r* = 0.51 and 0.56, respectively, *p* < 0.01). Adaptability was negatively correlated with COVID-related PTSD symptoms, supporting Hypothesis 1 (*r* = −0.43, *p* < 0.01). Perceived organizational support was also significantly negatively correlated with COVID-19-related PTSD symptoms, supporting Hypothesis 2 (*r* = −0.30, *p* < 0.01). Family support was not significantly correlated with COVID-19-related PTSD symptoms and Hypothesis 3 was not supported.

**TABLE 1:** Means, standard deviations, and correlations with confidence intervals (*N* = 277).

Variable	*M*	*SD*	1	2	3
1. Adaptability	4.88	0.75			
2. Perceived organizational support	4.60	1.01	0.51**		
			[0.42, 0.59]		
3. Family support	3.88	0.69	0.56**	0.49**	
			[0.47, 0.63]	[0.39, 0.57]	
4. COVID-related PTSD symptoms	2.94	0.79	–0.43**	–0.30**	–0.00
			[–0.52, –0.33]	[–0.41, –0.19]	[–0.12, 0.12]

*M and SD are used to represent mean and standard deviation, respectively.*

*Values in square brackets indicate the 95% confidence interval for each correlation.*

*The confidence interval is a plausible range of population correlations that could have caused the sample correlation (Cumming, 2014).*

** indicates p < 0.01.

The main effects of adaptability, perceived organizational support, and family support were examined first in Model 1 (top of Table 2). Results show a negative relationship between adaptability and COVID-related PTSD symptoms (*b* = −0.54, *p* < 0.01) and perceived organizational support and COVID-related PTSD symptoms (*b* = −0.19, *p* < 0.01). But the regression analysis also revealed an unexpected positive relationship between family support and COVID-related PTSD (*b* = 0.46, *p* < 0.01). The absence of this relationship in Table 1, and the multicollinearity of the predictor variables suggests a suppressor effect (Cohen and Cohen, 1983). Together the main effects of adaptability, perceived organizational support, and family support accounted for 28.8% of the variance in COVID-19-related PTSD symptoms.

**TABLE 2:** Regression results predicting COVID-related PTSD symptoms (*N* = 277).

Predictor	*b*	*b* 95% CI [LL, UL]	*sr* ^2^	*sr*^2^ 95% CI [LL, UL]	Fit
**Model 1**					
(Intercept)	4.67**	[4.11, 5.23]			
Adaptability	−0.54**	[−0.68, −0.41]	0.16	[0.09, 0.24]	
Perceived organizational support	−0.19**	[−0.28, −0.09]	0.04	[0.00, 0.08]	
Family support	0.46**	[0.32, 0.60]	0.11	[0.04, 0.17]	
					*R*^2^ = 0.288**
					95% CI [0.20, 0.36]
**Model 2**					
(Intercept)	3.06**	[2.97, 3.15]			
Adaptability	−0.54**	[−0.67, −0.41]	0.16	[0.09, 0.23]	
Family support	0.38**	[0.23, 0.52]	0.07	[0.02, 0.12]	
Perceived organizational Support	−0.12*	[−0.22, −0.01]	0.01	[−0.01, 0.03]	
Adaptability × Family support	−0.18*	[−0.34, −0.02]	0.01	[−0.01, 0.03]	
Adaptability × Perceived organizational support	−0.15**	[−0.26, −0.05]	0.02	[−0.01, 0.05]	
					*R*^2^ = 0.335**
					95% CI [0.24, 0.40]

*A significant b-weight indicates the semi-partial correlation is also significant.*

*b represents unstandardized regression weights.*

*sr^2^ represents the semi-partial correlation squared.*

*LL and UL indicate the lower and upper limits of a confidence interval, respectively.*

* indicates p < 0.05.

** indicates p < 0.01.

To examine the moderating effects of perceived organizational support and family support on COVID-19-related stress, we added interaction terms created with mean-centered variables in Model 2 (bottom of Table 2). In addition to the significant main effects of adaptability, family support, and perceived organizational support, both interactions were significant (*b*-values are −0.18 and −0.15 for family support and perceived organizational support, respectively). The interactions accounted for a significant additional 4.7% of variance in COVID-19-related PTSD symptoms above the main effects, *F*(2,271) = 9.58, *p* < 0.001.

**FIGURE 3:**
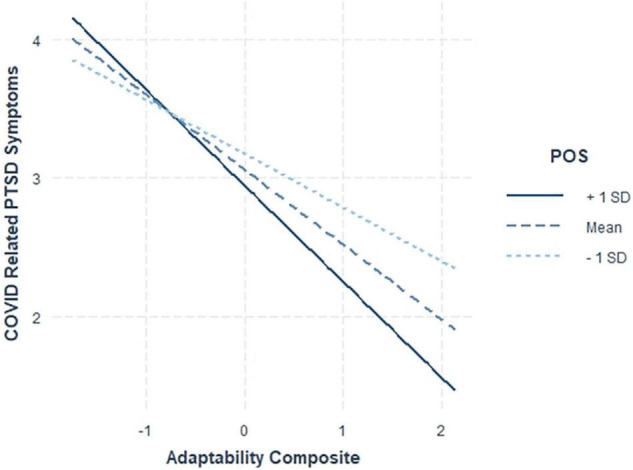
Interaction between adaptability and perceived organizational support (POS) on COVID-19-PTSD symptoms.

**FIGURE 4:**
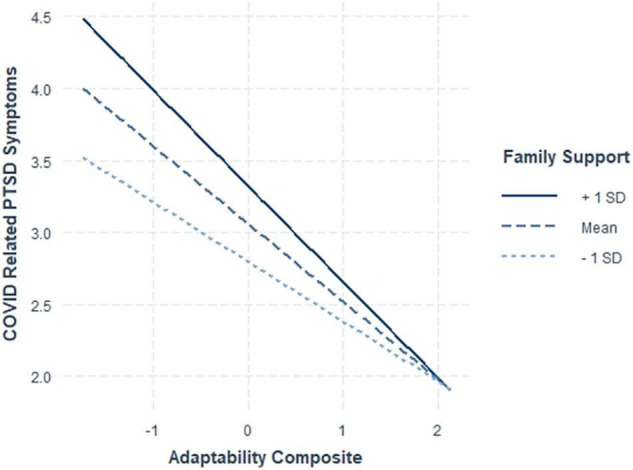
Interaction between adaptability and family support on COVID-19-PTSD symptoms.

To aid in interpretation, interactions were plotted using one standard deviation above and below the mean and the mean level of family support (all predictors centered) and results are shown in Figure 3 for perceived organizational support and Figure 4 for family support. Contrary to Hypothesis 4, which stated that nurses lower in adaptability would benefit more from perceived organizational support, we found that the level of perceived organizational support did not seem to matter for nurses relatively lower in adaptability, but perceived organizational support was helpful in mitigating PTSD symptoms for nurses relatively higher in adaptability.

For family support, our findings were also contrary to our expectations that family support would be most beneficial for nurses relatively lower in adaptability (Hypothesis 5). Figure 4 illustrates that nurses relatively low in adaptability reported more PTSD symptoms when they had more family support (rather than less). For nurses higher in adaptability, family support did not seem to matter as much for the reporting of PTSD symptoms. That is, adaptable nurses were less likely to experience PTSD symptoms regardless of their level of family support.

## Discussion

The purpose of this study was to examine the effect of adaptability, family support, and perceived organizational support on stress reactions to the COVID-19 pandemic in a sample of *N* = 277 Texas-based frontline nurses working in COVID-19 units. We also examined whether the relationship between adaptability and PTSD symptoms was moderated by the level of support that nurses receive in their organizations and families. We found, as expected, that nurses who were higher in adaptability and who perceived support from their organization reported fewer PTSD symptoms related to COVID-19. Furthermore, we found that perceived organizational support moderated the relationship between adaptability and COVID-19-related PTSD symptoms such that organizational support helped nurses cope when nurses were already relatively adaptable. When nurses were not adaptable, however, perceived organizational support did not make any difference in the reporting of COVID-19-related PTSD. This result was contrary to our prediction that perceived organizational support would be particularly helpful for nurses lower in adaptability. Indeed, research suggests that organizational support is positively related to important organizational outcomes such as job satisfaction and job performance (Erdogan and Enders, 2007; Zhou et al., 2015) and is an important buffer against burnout and strain that may negatively affect employee health (Lecca et al., 2020). Thus, it is interesting to consider why we did not find a buffering effect of perceived organizational support for relatively low-adaptive nurses in this study. One reason is that nurses lower in adaptability may have been so overwhelmed with the pandemic conditions such that organizational support did not provide any relief. Although interesting to consider, we know of no research that has examined the effect of perceived organizational support in conditions as extreme as a pandemic. Additional research is necessary to evaluate the boundary conditions of worker adaptability.

Our findings relative to family support were also unexpected. We did not find that family support reduced the likelihood that nurses reported COVID-19-related PTSD symptoms, but the opposite. Nurses who reported more family support were likely to report more COVID-19-related PTSD symptoms after accounting for other variables. Moreover, we found an interaction between nurse adaptability and family support such that nurses low in adaptability that perceived relatively more family support were *more* likely to report COVID-related PTSD symptoms compared with low adaptable nurses with relatively less family support. For nurses higher in adaptability, family support did not make as much difference for reporting COVID-related PTSD symptoms.

Our results regarding family support are indeed surprising, but it is helpful to situate our findings in the context of prior research showing that family support reduces negative outcomes like burnout for nurses (e.g., Kim and Choi, 2015). One potential reason for our divergent results is that burnout is conceptually different from PTSD symptoms in that burnout is job-related whereas PTSD symptoms are more globally related to a nurse’s overall mental health. Moreover, it may be that nurses who have close-knit families (i.e., nurses who are likely to perceive family support) may be more vulnerable to COVID-19-related PTSD symptoms because they are worried about those families, particularly when they are relatively low in adaptability. Conversely, nurses who are adaptable might learn how to accept and manage change and handle emergencies or crises at work and are potentially better able to put worry about family out of their minds (Pulakos et al., 2000, 2002).

For many nurses, it is likely that purposely staying away from home to avoid family exposure of infection may amplify feelings of social isolation (Li M. et al., 2020) which in turn may exacerbate PTSD symptoms. Indeed, research suggests that the greatest concern of nurses and other frontline care providers is protection of their family members, especially those nurses with younger children, those with special needs, or elderly extended family living in the home (Moderato et al., 2021). This interpretation of our findings aligns with others who found nurses were more stressed during the COVID-19 pandemic due to concerns about their family members becoming ill (Arnetz et al., 2020). Further, in a meta-analysis of prevalence of symptoms among healthcare providers working on the frontline during epidemics/pandemics, it was reported that 60% had concerns about transmitting the virus to family members (Busch et al., 2021).

Sasangohar et al. (2020) examined frontline health workers in a high-volume intensive care unit during the COVID-19 pandemic and observed them “emotionally breaking down mainly due to the added pressure to choose between family responsibilities and their inner sense of duty toward patients” (Sasangohar et al., 2020, p. 106). Nonetheless, to tease apart whether the negative effect of family support on COVID-19-related stress symptoms is a function of the support that a family provides (as measured in this study) or the mere existence of a family at all, we would need to have measured family status. Unfortunately, we did not ask nurses to report their family status. As such, examining whether our effects are a function of the existence of a family, or the support from family is unknown and an important area of future research.

### Limitations

As with all research, there are limitations to the present study. First, there are limitations with the single-source cross-sectional nature of the study, which limits our ability to establish causal relationships, increases the likelihood of common method variance, and makes it impossible to know the psychological status of the sample of nurses before the pandemic. Unfortunately, a more elaborate research design (multi-time point, informant raters) was not possible given the limited availability of nurses during the COVID-19 pandemic, but these limitations should be kept in mind when interpreting the results of this study.

We also collected data in a single state in the United States and we did not include a control group. Although this may limit the generalizability of our findings somewhat, a focus on a single state permits us to more clearly understand how the pandemic was influencing nurses in that state during the time of data collection (spring, 2020). Notably, Texas is home to over 51 Magnet hospitals, the highest number of any state in the United States.^1^ As such, results of this study support the “best case scenario” for nurses practicing during COVID-19 in the United States. Also, the sample of nurses who participated in the current study is relatively younger than the average nursing workforce in the United States of 51 years (NCSBN), and so our results may not generalize to all nurses. Finally, only a small number of nurses who had access to the survey completed it. Although a relatively low response rate is not surprising given that during the time the study was conducted nurses were dealing with unprecedented stress at work. Nonetheless, the low response rate should be considered when interpreting the results of this study.

### Implications

The implications of this study point to the importance of adaptability and perceived organizational support for nurses. As such, we recommend that healthcare leaders provide organizational support for the nursing workforce. Supervisors’ social support, a form of organizational support, is an essential predictor of psychosomatic distress, a symptom of PTSD, in nurses working in high-stress environments (Adriaenssens et al., 2011; Lecca et al., 2020). Highly engaged, participative leadership facilitates discussing problems as a group, sharing and processing ideas, making decisions, implementing the decision, and empathetic leadership involves understanding the nurse’s needs and being aware of the thoughts and feelings of the nursing staff (Adriaenssens et al., 2011; Zhou et al., 2015). Teamwork and morale-building activities go a long way to show support. Developmental feedback, job rotations, training, and rewards, can also improve the perception of organizational support (Cullen et al., 2014; Lecca et al., 2020).

For adaptability, recommendations to reduce stress are shorter staffing shifts, such as 8-h rather than 12-h, and supporting fatigue countermeasures (e.g., requiring more frequent breaks with healthy foods, drink, and a place for rapid-rest periods). Hospitals in Texas have offered full-time, part-time and relief nurses stay-bonuses. In addition, some leadership teams were offering large sign-on bonuses to entice nurses to return to the bedside. Middle management should reinforce break practices that help unwind and reset from cognitive overload and combat fatigue. Nurses need sleep resources, a quiet place to rest, hospitals to provide necessary resources such as places to shower, supply scrubs, and toiletry items for overnight stays (Sagherian et al., 2020).

According to Levin (2019), public health officials can administer psychological first aid with respectful rapport, triaging psychological needs, teaching guided imagery, mindfulness, deep breathing, and progressive muscle relaxation. Counselors to assist with normalizing anger to decrease anger-driven behaviors can be therapeutic to families and co-workers. Further safety plans to reduce the risk of suicide should be proactive in assessing and providing interventions due to acute anxiety, stress, bereavement, and multiple losses (Levin, 2019). Proving crisis chaplains from faith-based communities offer spiritual guidance to affected staff and can be a great comfort, outlet, and support in fearful, exhausting, uncertain times (Levin, 2019). Barriers are present to seeking help, but organization culture should lend itself to a safety culture and not a punitive mindset. Barriers to seeking care include stigmatization, such as being considered to be infectious, being able to handle oneself and handle all areas of life, and not allowing to feel safe to grieve the exposure to multiple deaths and feeling the burdened compounded by multiple deaths in a shift, separation of family, and isolation. US nurses have experienced significant loss of colleagues, family members, patients, and normalcy to COVID-19. We must support and help nurses move forward, recognizing the importance of individual adaptability, family support, and organizational support on PTSD outcomes. If left unaddressed, according to Levin (2019) emotional needs and psychological wounds of healthcare providers can reverberate long.

Interpersonal adaptability can be strengthened by being flexible and open-minded when dealing with others, listening to and considering others’ viewpoints and opinions and altering one’s own opinion when it is appropriate, being open and accepting of negative or developmental feedback, working well and developing effective relationships with highly diverse personalities, demonstrating keen insight of others’ behavior and tailoring one’s own behavior to persuade, influence, or work more effectively with them (Pulakos et al., 2002). Work stress-oriented adaptability can be promoted by remaining composed when faced with difficult circumstances or a highly demanding workload or schedule, not overreacting to unexpected news or situations, managing frustration by directing effort to constructive solutions rather than blaming others, demonstrating resilience and the highest levels of professionalism in stressful circumstances, and acting as a calming and settling influence to whom others look for guidance (Pulakos et al., 2002). Lastly, uncertainty-oriented adaptability can be improved through knowledge acquisition by learning to take effective action when necessary without having to know the total picture or having all the facts at hand, readily and easily changing gears in response to unpredictable or unexpected events and circumstances, effectively adjusting plans, goals, actions, or priorities to deal with changing situations, imposing structure for self and others that provide as much focus as possible in dynamic situations, not needing things to be black and white, and refusing to be paralyzed by uncertainty or ambiguity (Pulakos et al., 2002).

For family support, nurses with families will experience even more stress than those without. Healthcare leaders should consider the impact of family support on PTSD severity. Additional support that families receive will determine how well a family will adapt to a crisis (LoBiondo-Wood, 2003). Although we did not measure the impact of family members of nurses working in acute care environments during the pandemic, reinforcing coping strategies with the whole family is a family-centered approach that appears worthwhile.

## Conclusion

During the early months of the outbreak of COVID-19 pandemic in the United States, there is limited research due to the priority being patient care. This study is one of the first studies to evaluate the response of nurses to the pandemic from May to July 2020. Longitudinal studies are needed to evaluate the long-term mental health effects of PTSD on nurses, who experience greater risk of exposure to the virus due to the large amount of time spent caring for COVID-19 patients, and greater exposure to death, ethical dilemmas and suffering. Hospital administrators can improve the situation by being attentive to psychological, physical, spiritual, and psychosocial needs of hospital employees, especially front-line COVID-19 staff to reduce stress and prevent burnout. Mental distress can be lessened by providing means for nurses to communicate with their families, provide local housing near the hospital to allow for limited exposure to their families, showering facilities after shift, and disposable or clean scrubs to reduce exposure. Organizational support is key to reducing the risk of PTSD in frontline care givers.

## Data Availability Statement

The datasets presented in this article are not readily available because work related to the dataset is ongoing. Requests to access the datasets should be directed to the corresponding author.

## Ethics Statement

The studies involving human participants were reviewed and approved by Sam Houston State University, Rice University, and University of Texas Health Science Center. Written informed consent for participation was not required for this study in accordance with the national legislation and the institutional requirements.

## Author Contributions

MC: writing of proposal, IRB submission, study design, and data collection. MB: study design, statistical analysis, and writing of manuscript. SB and LB: study design and writing of manuscript. All authors contributed to the article and approved the submitted version.

## Conflict of Interest

The authors declare that the research was conducted in the absence of any commercial or financial relationships that could be construed as a potential conflict of interest.

## Publisher’s Note

All claims expressed in this article are solely those of the authors and do not necessarily represent those of their affiliated organizations, or those of the publisher, the editors and the reviewers. Any product that may be evaluated in this article, or claim that may be made by its manufacturer, is not guaranteed or endorsed by the publisher.

## Footnotes

1
https://resources.nurse.com/magnet-hospitals-in-your-state
